# Elucidating the Thermal Properties of Partially Chlorinated Graphene Using Molecular Dynamics Simulations

**DOI:** 10.1021/acs.jpcc.5c04046

**Published:** 2025-09-17

**Authors:** Javier Varillas, Martin Kalbáč

**Affiliations:** † Department of Low-Dimensional Systems, J. Heyrovský Institute of Physical Chemistry, Czech Academy of Sciences, Dolejškova 2155/3, 182 23 Prague 8, Czech Republic; ‡ Department of Ultrasonic Methods, Institute of Thermomechanics, Czech Academy of Sciences, Dolejškova 1402/5, 182 00 Prague 8, Czech Republic

## Abstract

We investigated thermal transport in partially chlorinated graphene (PCG) via molecular dynamics (MD) simulations using a hybrid force field (h-FF) tailored for chlorinated systems. The h-FF integrates a Tersoff-type potential for C–C interactions with pairwise Morse and Lennard-Jones models for bonded C–Cl and nonbonded C–Cl/Cl–Cl interactions, respectively, including atomic charge equilibration. The Morse potential is fitted to reproduce key chemical and physical properties of C–Cl covalent bonds, while h-FF calibration aims at binding energies and bond lengths predicted by density functional theory. We relaxed suspended and supported PCG sheets with ∼1.5–25% Cl content at 300 K, confirming their thermal stability. To assess the thermal properties of PCG, we analyzed the vibrational modes captured by the simulations and compared the phonon dispersion with that of single-layer graphene (SLG). In PCG, the highest optical modes flattened and acoustic-mode frequencies downshifted due to enhanced phonon scattering, reducing thermal transport. Nonequilibrium MD simulations confirmed a marked reduction in thermal conductivity with increasing Cl content, dropping by ∼70% at ∼1% Cl content and by ∼98% at ∼25% Cl content. The h-FF model enables efficient, accurate predictions of thermally relaxed PCG sheets, offering key insights into their thermal behavior vis-à-vis SLG.

## Graphical abstract

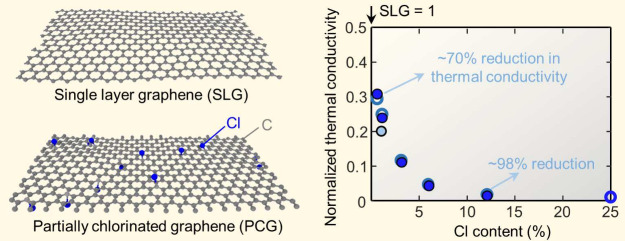

## Introduction

All-carbon single-layer graphene (SLG), the quintessential two-dimensional (2D) material, has the strongest mechanical performance measured to date.^1^ The combination of its honeycomb lattice symmetry with the sp^2^ hybridization of the C–C bonds fundamentally determines the electrothermal properties of SLG, including its intrinsic zero bandgap and limited phonon scattering. However, its high electrical conductivity hampers the potential implementation of SLG-based systems in electronic devices that require nonzero bandgap. Theory and experiments have shown that the thermal conductivity of suspended SLG can reach ultrahigh values as much as about ∼5000 W/(mK),^2^ seemingly converging to ∼3000 W/(mK),^3^ which is still one other of magnitude larger than that of highly conductive metals.

Graphene chemical functionalization modifies SLG to tune its properties, where functional atoms (or molecules) bond to the graphene lattice, creating local sp^3^ hybridization states and opening a bandgap, enabling the use of graphene-based materials in electronics. Graphene halogenation—sp^3^-hybridized sites introduced by bonded halogen atoms (such as F, Cl, Br, and I^4^) to the surface of graphene—is among the most studied functionalization techniques. It provides an effective approach for tailoring the thermal and electronic properties of graphene through phonon-dispersion and bandgap engineering, respectively, while preserving the exceptional mechanical strength of sp^2^ C–C bonds.^5^

Although graphene fluorination is the most extensively studied halogenation technique, graphene chlorination has recently raised interest in the 2D materials community because of the high stability of chlorinated graphene systems^6^ along with its p-type doping tunability^7^ and promising bandgap engineering (as theoretical predictions^5,8^ and experimental measurements^9^ reported a bandgap close to that of Si). These new insights make chlorinated graphene a desirable candidate for diverse technological applications, ranging from solar cells,^8^ energy storage,^10^ white-light emission,^11^ rewritable photodetection,^12^ and antibacterial nanomaterials.^13^

Chemical chlorination of graphene has been reported using various experimental means, including photochemical^6,7,14^ electrochemical,^15^ laser-assisted,^12^ and plasma-based^10,16–18^ techniques. Although fully chlorinated graphene (chlorographene) has been theoretically predicted to be a stable configuration,^5^ the Cl content in experimental chlorinated graphene samples is generally reported as ∼1–30% (notably, high Cl contents of up to ∼45% have been recently found in supported chlorinated graphene samples^12,16^ ).

Despite these experimental and theoretical efforts, the thermal transport in chlorinated graphene systems and the effect of Cl concentration on the lattice dynamics (i.e., phonon dispersion) of graphene remain to be assessed. Extensive SLG research has provided fundamental insights into its phonon behavior, which fundamentally confers its ultrahigh thermal conductivity. While the presence of C–Cl bonds is expected to play a crucial role in acoustic phonon motion, theoretical studies are currently lacking for partially chlorinated graphene (PCG). Elucidating the phonon modes is then essential for understanding the impact of functionalization on thermal transport across chlorinated graphene sheets.

Molecular dynamics (MD) simulations are widely used to study thermal transport in 2D materials due to their ability to model atomic-level interactions while capturing anharmonic effects (i.e., high-order phonon scattering), which are critical features for heat conduction of graphene-based systems. Unlike first-principles methods such as density functional theory (DFT), which are computationally prohibitive when it comes to simulating systems involving thousands of atoms, MD enables the study of much larger systems that can contain millions of atoms, reaching micrometer-scale lengths.^19^ Accurate and computationally efficient interatomic potentials are paramount for investigating thermal transport over length scales that exceed the phonon mean free path.^20^

In this study, we investigate the thermal transport in PCG systems with Cl content ranging from ∼0.5% to ∼25%. To this end, we implement a computationally efficient, hybrid force field (h-FF) for large-scale MD simulations. The h-FF model combines a Tersoff-type potential for the C–C interactions with pairwise Morse and Lennard-Jones potentials for the bonded C–Cl and nonbonded C–Cl/Cl–Cl interactions, respectively. We calibrate the h-FF model to fit various chemical and physical properties of the C–Cl bonds based on previous DFT results, including binding energies and bond distances. Using this model, we elucidate the thermal properties of PCG by analyzing the phonon modes explicitly derived from MD simulations at 300 K. Additionally, we conduct nonequilibrium molecular dynamics (NEMD) simulations to establish the relation between the in-plane thermal conductivity and Cl content in PCG. The present h-FF model is, up to date, the first MD force field tailored to chlorinated graphene systems.

## Computational Methods

We run molecular dynamics (MD) simulations using the open-source LAMMPS package.^21,22^ Atomistic visualization is conducted using the OVITO PRO package.^23^ The computational time step, *dt*, is set (unless stated otherwise) to 0.5 fs. The equations of motion are integrated using the velocity Verlet algorithm. Prior to MD thermalization, the sheets are systematically subjected to energy minimization using the steepest descent method.

In this section, we describe the classical, hybrid force field employed to simulate partially chlorinated graphene (PCG), including the methods for computing long-range electrostatic forces and atomic charge equilibration. The construction of suspended and substrate-supported PCG systems is also explained. Finally, we detail the calibration of the force field, aimed at reproducing the binding energies and bond lengths reported in density functional theory (DFT) studies of chlorinated graphene systems.

Supplementary files include the necessary LAMMPS input files to conduct a PCG simulation using the h-FF potential, following the methodology described in this section.

### Classical Force Field for MD Simulations of PCG Systems

To reproduce the physical features of sp^2^- and sp^3^-type bonds in chlorinated graphene, we utilize a hybrid force field (h-FF), hereafter the h-FF model, which combines a Tersoff-type potential for C–C interactions with pairwise Morse and Lennard-Jones potentials for bonded C–Cl and nonbonded C–Cl/Cl-Cl interactions, respectively, while incorporating atomic charges.

We adopt the optimized Tersoff (opt-Tersoff^24^) potential to describe the C–C interactions. This reactive-type potential is the most accurate force field for simulating the thermal properties of both single- and multilayer graphene sheets, outperforming other bond-order potentials such as REBO and AIREBO.^25^

The covalent-bonded C–Cl pairs are described by the pairwise interaction energy, *E*(*r*_*ij*_), through

1 $$E\left(r_{ij}\right)=E_{\mathrm{nb}}\left(r_{ij}\right)+E_{b}\left(r_{ij}\right)$$

where *r*_*ij*_ is the distance between two interacting atoms *i* and *j*, and subscripts ‘b’ and ‘nb’ refer to the bonded and nonbonded pairwise interaction type, respectively.

We define *E*_nb_(*r*_*ij*_) as the combination of the short-range dispersion and long-range electrostatic forces, modeled by the 12–6 Lennard-Jones (LJ) and the Coulomb (Cb) potentials, respectively. Thus,

2 $$E_{\mathrm{nb}}\left(r_{ij}\right)=E_{\mathrm{LJ}}\left(r_{ij}\right)+E_{\mathrm{Cb}}\left(r_{ij}\right)$$

where

3 $$E_{\mathrm{LJ}}\left(r_{ij}\right)=4\epsilon \left[{\left(\frac{\sigma }{r_{ij}}\right)}^{12}-{\left(\frac{\sigma }{r_{ij}}\right)}^{6}\right]$$

with σ and ϵ being the LJ potential parameters, representing the zero-energy distance and the potential depth, respectively; and

4 $$E_{\mathrm{Cb}}\left(r_{ij}\right)=k_{e}\frac{q_{i}q_{j}}{r_{ij}}$$

with *k*_e_ being Coulomb’s constant (*k*_e_ ≈ 8.9875 × 10^9^ Nm^2^C^−2^) and *q*_*i*_ and *q*_*j*_ the charges on atoms *i* and *j*, respectively. Note that the attractive part in *E*_nb_(*r*_*ij*_) is captured by the – 1/*r* ^6^ term in the LJ potential and the Coulombic – 1/*r* interactions (when *q*_*i*_ and *q*_*j*_ charges are of opposite sign). The multilevel summation method (MSM) computes the electrostatic forces, using nested interpolation which avoids fast Fourier transformations in the calculations, boosting computational efficiency while maintaining accuracy.^26^ (Unlike the Ewald summation, nonperiodic boundary conditions are permitted within the MSM framework.)

In addition, *E*_b_(*r*_*ij*_) (bonded C–Cl pairs) is modeled by the Morse potential, which has been proven to be an accurate and efficient pairwise function for covalent-type bonds in various MD systems, including graphene^27,28^ and carbon nanotubes.^29^ We adopt the Morse curve implemented in LAMMPS via the ‘pair_style morse/smooth/linear’ command,^30^ defined as

5 $$E_{b}\left(r_{ij}\right)=D_{0}\left[e^{-2\alpha \left(r_{ij}-r_{0}\right)}-2e^{-\alpha \left(r_{ij}-r_{0}\right)}\right]$$

where *D*_0_ determines the depth of the Morse potential well, *r*_0_ is the equilibrium bond distance, and α tunes the shape of the potential (i.e., decreasing values of α broaden the well). Note that *E*_b_(*r*_*ij*_) > 0 for *r*_*ij*_ < *r*_c_, where *r*_c_ is the cutoff for the potential, allowing C–Cl bond dissociation without relying on a bond-order model.^27^

The interatomic forces between nonbonded C–Cl and Cl–Cl interactions are calculated using *E*_nb_ from eq 2, where the LJ parameters for Cl–Cl interactions are σ = 3.4 Å and ϵ = 0.0130 eV (Table S1 in ref 31). We set the LJ parameters for C–Cl interactions (in PCG) to σ = 1.8 Å and ϵ = 0.0032 eV. The cutoff distance for the short-range LJ terms is set to 2.5σ, while the cutoff for the long-range Coulombic forces is set to 15 Å, which renders a good accuracy for MSM-computed forces.

The C–Cl bonds are assumed to have polar covalent character, containing unequal atomic charges. The more electronegative atom (Cl with electronegativity χ_Cl_ = 3.16 in Pauling scale) gains a partial negative charge, and the less electronegative atom (χ_C_ = 2.55) gains a partial positive charge. (Although several experimental reports confirmed the presence of C–Cl covalent bonds in various chlorinated graphene systems,^14,16,17^ the charge levels produced in bonded C–Cl pairs remain to be addressed for graphene chlorination.) The electrostatic energy of the PCG systems is minimized using the charge equilibration (QEq) method,^32^ based on Sanderson’s postulate, which states that electron density transfers between atoms so that electronegativity at all atomic sites is the same at equilibrium. The atomic charges are calculated using matrix inversion to solve electronegativity equalization, satisfying charge conservation. For the QEq calculations, we adopt the point charge model.

### The As-Built SLG and PCG Systems

We build MD domains that contain flat, pristine sheets of suspended single-layer graphene (SLG), where the initial C–C bond length is set to 1.4388 Å.^24^ Additionally, we induce random-site chlorination in the SLG cells by introducing functionalized C atoms bonded to Cl atoms. We limit the chlorination of C sites to only one Cl atom.^5^ Also, the presence of same-side Cl atoms bonded at nearest-neighbor C sites is not allowed, as this configuration is expected to be unstable in halogenated graphene systems with charged covalent bonds. The PCG systems have periodic boundary conditions on the lateral walls of the MD domains, whereas the top and bottom boundaries are nonperiodic and fixed.

We construct both suspended (double-sized chlorination) and supported (single-sized chlorination) PCG sheets with low-to-moderate Cl content, *X*_Cl_, ranging from ∼0.5% to ∼25%. The as-built C–Cl bonds have an angle of 90° (i.e., normal to the plane forming the as-built SLG) and a length that corresponds to the lowest-energy (ground-state) bond distance, forming (single-layer) PCG. The suspended PCG sheets (Supplementary Figure S1) are constructed under the lowest-energy C–C bond length, $d_{C-C}^{0}$ , which is determined by running molecular static (MS) simulations. In these simulations, the sheets are expanded (or stretched) along the *x* and *y* directions, varying the C–C bond distances from 1 Å to 3 Å; see Supplementary Figure S2a.

Below the supported sheets, we construct a cuboidal-shaped monocrystalline Si substrate that matches the *x* and *y* dimensions of the top PCG sheet, which adheres to the substrate’s top surface through weak van der Waals (vdW) forces. The thickness of the substrate is ≈ 2 nm. To define the Si–Si interactions, we adopt the modified Tersoff potential by Pun and Mishin,^33^ which accurately reproduces the thermal properties of various crystalline Si structures. The Si substrate is made of a (001)-oriented, diamond-structured Si crystal with a lattice parameter of 5.434 Å.

Following the first-principles calculations by Gao et al.,^34^ who demonstrated that the vdW interaction is the predominant adhesion mechanism at the interface between SLG and Si-based substrates, we model the Si–C interactions using the LJ potential (eq 3), which effectively describes interfacial vdW forces. For the Si–C (sheet-substrate) interactions, we use the LJ parameters σ = 2.95 Å and ϵ = 0.0221 eV, which render similar sheet-substrate adhesion levels to those measured between SLG and Si-based substrates. We estimate the SLG/Si-substrate adhesion predicted in our simulations through the continuum model proposed by Zhu et al.^35^ The LJ parameters are fitted to yield the experimental cohesive energy (∼0.45 J/m^2^ ^36^). The initial separation between the PCG sheets and the Si substrate satisfies the distance (2.8 Å) that ensures the minimum energy configuration; see the inset to Supplementary Figure S1b.

### Calibration of the Classical Force Field for PCG

We parametrize the Morse curve for C–Cl bonds (eq 5) to reproduce fundamental physical features of PCG systems, including the binding energy (BE) and bond length levels predicted by DFT calculations of Cl absorption on SLG.^37^

First, we determine the parameter α of the Morse potential by fitting the bond energy curve near *r*_0_ (cf. Figure 1 in ref 29). Due to the absence of theoretical results showing the C–Cl bond energy profile in chlorinated graphene systems, we fit α based on the bond energy curve that the reactive force-field (ReaxFF) potential by Singh et al.^38^ predicts for partially fluorinated graphene (PFG). Since α characterizes bond stiffness, associated with the optical-mode vibrations of the lattice,^29^ we assume that its value for C–Cl bonds in PCG is compatible with that of C–F bonds in PFG. This idea is supported by Raman spectroscopy measurements, which show similar G-mode peaks around 1525 cm^–1^ in both chlorinated and fluorinated graphene systems.^16,39^ The analysis yields α = 4.2 Å^–1^; see Supplementary Figure S3.

The energy-minimum distance of the Morse potential, *r*_0_, is fixed to the theoretical equilibrium C–Cl bond distances.^37^ We set the cutoff (dissociation) distance of the Morse potential, *r*_c_, to 3.3 Å, since C–Cl bonds are not expected to exhibit lengths greater than this value (which is the DFT-predicted dissociation distance for Cl atoms absorbed on graphene^37^). The Morse potential energy (and force) is 0 at *r*_c_, enabling bond dissociation when *r* > *r*_c_, as demonstrated in previous MD studies.^27,40^

We determine the potential depth, *D*_0_, whose value yields consistent BE levels vis-à-vis DFT data. To this end, we perform 200-ps *NPT* thermalizations using MD cells of size 7 nm × 7 nm × 10 nm (in *x*, *y*, and *z* directions, respectively) at *T* = 10 K for *X*_Cl_ = ≈ 6.25%, 12.5%, and 25%. (Note that energy calculations from low-temperature MD simulations enable a direct comparison with ground-state energy values predicted by DFT.) Further details on the thermostat and barostat used in the *NPT* simulations are provided later in the text.

The procedure employed to fit the parameters of the Morse potential for PCG starts from an initial guess for parameter *D*_0_, which corresponds to the DFT-derived BE at a given *X*_Cl_. We calculate the BE (of Cl adatoms absorbed on SLG) by recourse to the following formula:^41^

6 $$\mathrm{BE}=\left(E_{\mathrm{PCG}}-E_{\mathrm{SLG}}\right)/N_{\mathrm{Cl}}$$

where *E*_PCG_ and *E*_SLG_ are the potential energies of the PCG and SLG systems, respectively; and *N*_Cl_ stands for the number of absorbed (bonded) Cl atoms on SLG forming PCG. *E*_PCG_ is the average value of the system’s potential energy obtained during the *NPT* thermalization, whereas *E*_SLG_ is equally computed from the SLG simulation. Potential energies are averaged over 100 ps, as illustrated in Supplementary Figure S4 and the inset to Supplementary Figure S5 for 300 and 10 K, respectively. Note in these figures that box dimensions and energy in *NPT*-thermalized SLG and PCG sheets converge after 20–50 ps. Supplementary Figures S5 and S6 show the influence of *D*_0_ on the BE, the average charge of Cl atoms (*q*_Cl_), and the average charge difference between bonded C–Cl pairs (Δ*q*) obtained in relaxed PCG sheets. The lowest-energy C–C and C–Cl bond lengths (i.e., $d_{C-C}^{0}$ and *r*_0_, respectively) used to construct the PCG systems, along with the fitted Morse potential parameters, are provided in Table 1.

**Table 1:** Values for the Parametrized Morse Potential That Describes the Covalent C–Cl Bonds under Various PCG Configurations (Suspended Sheets, Double-Sided Chlorination)^a^

*X*_Cl_ (%)	*D*_0_ (eV)	*r*_0_ (Å)	*d*_C–C_^0^ (Å)
0.5–9	1.38	2.00	∼1.45
9–18	1.19	1.95	∼1.46
18–25	1.68	1.90	∼1.47

a The *d*_C–C_^0^ values were elucidated from MS simulations (Supplementary Figure S2). *r*_0_ is set to the DFT-predicted C–Cl bond lengths.^37^ The fixed Morse parameters are α = 4.2 Å^–1^ (Supplementary Figure S3) and *r*_c_ = 3.3 Å. We choose the *D*_0_ values that yield the BEs anticipated by DFT^37^ (calibration results in Supplementary Figures S5 and S6). See the main text for further details.

Figure 1a shows the C–Cl bond energy generated by the h-FF model (eqs 1–5) as a function of the C–Cl distance, *r*, under distinctly charged C–Cl bonds (left: Δ*q* = −0.03 e; right: Δ*q* = −0.3 e; 1.0 e represents the charge of a proton). Notice in this figure that the attractive part of the h-FF becomes charge-dominant for large Δ*q* levels. The energy profiles are evaluated using the C_8_Cl supercell depicted in Figure 1b.

**Figure 1:**
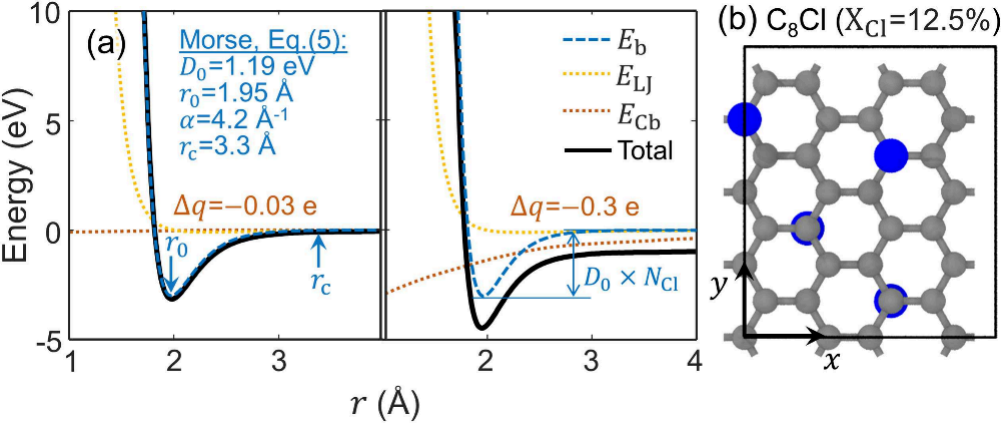
Pair-wise interaction in bonded C–Cl pairs. (a) Energy profile with varying bond distance, *r*, using the C_8_Cl supercell depicted in (b). The potential energy levels are computed using two values of charge difference (Δ*q*) between bonded C–Cl pairs: Δ*q* = −0.03 e (left) and −0.3 e (right). (b) C_8_Cl (*X*_Cl_ = 12.5%) supercell with double-sided chlorination, containing 32 C atoms (gray) and 4 Cl atoms (blue).

For the QEq calculations, we adopt the electronegativity (χ) and chemical hardness (η) values for isolated C and Cl atoms proposed by Verstraelen et al.,^42^ who calibrated these parameters for efficient and accurate charge equilibration using 500 organic compounds. Supplementary Figure S7 shows the impact of varying χ and η on the statistical values of BE, Δ*q*, and *q*_Cl_, as obtained from MD thermalizations of PCG with *X*_Cl_ ∼ 6%. This analysis justifies the chosen values of χ and η, which yield stable spatial distributions of atomic charges in our MD PCG systems.

Lastly, we assess the resulting BEs (using the parametrized Morse potential) in 7 nm × 7 nm PCG sheets at *T* = 10 and 300 K, with *X*_Cl_ ranging from ∼3% to ∼25% (double-sized chlorination). Figure 2a shows the resulting BEs with varying *X*_Cl_ (extracted from relaxed PCG sheets), aligning with the DFT-predicted BE values.^37^

**Figure 2:**
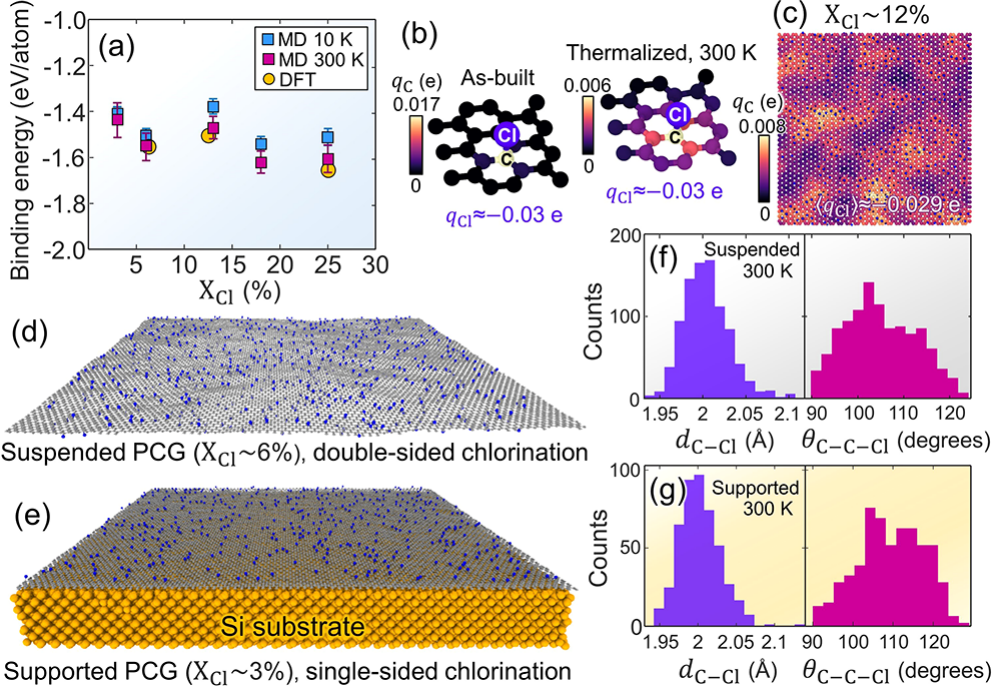
Binding energy (BE) and C–Cl bond properties in PCG sheets. (a) Calculated BEs as a function of Cl content, *X*_Cl_. DFT-estimated BE values are from ref 37. (b) Atomic charge distribution of an isolated C–Cl bond from as-built charge-neutral (left) to thermalized (right) states. (c) Spatial charge distribution of the C atoms in suspended PCG (*X*_Cl_ ∼ 12%) extracted from a 10 nm × 10 nm central slab. ⟨*q*_Cl_⟩ expresses the average value of *q*_Cl_. MD snapshots of (relaxed) suspended and supported PCG sheets are given in (d, e), respectively. The corresponding distributions of C–Cl bond lengths (*d*_C–Cl_) and angles (θ_C–C–Cl_) are plotted in (f, g). Cl atoms are colored in blue in (b–d, f), while the orange particles in (e) depict the Si atoms of the substrate.

## Results and Discussion

In this work, we primarily focus on assessing the thermal properties of PCG sheets, as predicted by the h-FF model tailored for MD simulations of chlorinated graphene systems. While DFT calculations suggest that *X*_Cl_ levels may saturate at 25% in PCG systems (patterned one-sided chlorination),^37^ usual levels of Cl concentration in real-life experimental samples vary from ∼1% to ∼25% (as in, e.g., refs 6, 14, 15, and 17 ). Therefore, we evaluate PCG systems with low-to-moderate *X*_Cl_, ranging from ∼0.5% to ∼25%.

In this section, we analyze the properties of relaxed C–Cl bonds across various PCG systems, considering the influence of substrate support and Cl content (*X*_Cl_). Also, thermal transport in suspended PCG sheets is examined through comparisons of phonon spectra and in-plane thermal conductivities relative to those of SLG sheets.

### Properties of C–C and C–Cl Bonds in Relaxed PCG Sheets

We conduct MD thermalizations of suspended PCG sheets (Supplementary Figure S1a), whose constituent particles sample the isothermal–isobaric *NPT* ensemble, allowing the *N*-particle system to reach thermodynamic equilibrium at target temperature *T* (= 300 K). The Nosé-Hoover (NH) thermostat controls the system’s temperature (which fluctuates around 300 K), using 3 NH chains and with the temperature damping parameter ‘Tdamp’ set to 100 *dt*.^43^ The NH barostat maintains the pressure, *P*, around 1 atm along the in-plane (*x* and *y*) directions; with the temperature damping parameter ‘Pdamp’ set to 1000 *dt*.^43^

Alternatively, we perform additional thermalizations of PCG sheets supported on a Si substrate, where the PCG particles follow the canonical *NVT* ensemble with the NH thermostat controlling *T*. The substrate is divided into two regions: an external, rigid region with unthermalized atoms, and an internal region with *NVT*-thermalized particles; see Supplementary Figure S1b. Since the volume of the MD domain (*V*) is fixed by the *NVT* ensemble in the supported cases, we built the PCG sheets using the *d*_C–C_ value determined in the relaxed suspended sheets. This approach eliminates potential prestraining effects during relaxation in volume-constrained simulations. All thermalization runs conducted for the analysis of the bond properties in relaxed PCG sheets span a time scale of 1 ns.

The h-FF model for PCG systems is approximately 4 times slower than the opt-Tersoff for SLG but remains about 20 times faster than the ReaxFF model for PFG;^38^ see Supplementary Figure S8. (Factoring in *dt*, still h-FF is about 8 times faster than ReaxFF, provided a small *dt* is required in a PCG simulation.) Since the contribution of pairwise interactions (Morse and LJ terms) is expected to be minimal due to their computational efficiency,^27^ the increased computational cost of PCG simulations compared to SLG primarily arises from the atomic charge calculations (i.e., long-range Coulombic forces via MSM and charge equilibration via QEq Although ReaxFF models are capable of capturing essential features in organic compounds, including bond formation and breaking, they are computationally expensive, which hinders their use for large-scale MD simulations.

According to the results from our 1 ns MD thermalizations, all the PCG systems modeled for this study exhibit thermal stability at 300 K as the total energy and spatial atomic charge distribution reach equilibrium; see Supplementary Figure S9. In the following, we describe the chemical and physical properties of the C–C and C–Cl bonds found in the relaxed (suspended and supported) PCG sheets, including atomic charges and the distribution of bond lengths and angles.

In our PCG sheets, positive charges accumulate in the vicinity of negatively charged Cl atoms, producing C–Cl bond polarization characterized by Δ*q* levels that range from ≈ – 0.027 e to ≈ – 0.022 e when *X*_Cl_ varies from ∼3% to ∼25%. Cl atoms exhibit atomic charges, *q*_Cl_, of ≈ – 0.03 e regardless of *X*_Cl_. Local charge equilibration is illustrated in Figure 2b for an isolated C–Cl bond, whereas Figure 2c shows the spatial distribution of C atom charges, *q*_C_, in a relaxed PCG sheet with *X*_Cl_ ∼ 12%. These results show that *q*_C_ locally concentrate when the Similar charge localization is found in PFG simulations using the ReaxFF model;^38^ see Supplementary Figure S9a,b.

Figure 2d shows the relaxed, suspended PCG sheet with *X*_Cl_ ∼ 6%, whose C–Cl bond length and angle distributions are drawn in Figure 2f. In the relaxed PCG sheets, the C–Cl pairs exhibit values of bond length, *d*_C–Cl_, that follow a Gaussian-like distribution characterized by an average value close to the imposed *r*_0_ and a variance of ∼1.5 Å. Notice that the statistical spread of *d*_C–Cl_ values roughly coincides with that from C–F bond length distributions in relaxed PFG (ReaxFF^38^); see Supplementary Figure S10c. These *d*_C–Cl_ distributions also align with those measured in aromatic organic compounds (cf. Figure S1 in ref 44).

These MD results suggest that the microstructure of relaxed, suspended PCG sheets with low *X*_Cl_ (<10%) exhibits minimal buckling. This is consistent with DFT calculations of single Cl atom absorption on the graphene lattice, which is expected to induce ultrasmall out-of-plane displacements (0.05 Å).^5^ With moderate *X*_Cl_ levels (10–25%), however, our suspended PCG sheets exhibit a mild decrease in mean C–C bond angles (Table 2), indicating slightly larger out-of-plane distortions around sp^3^ C sites. We would like to point out that the opt-Tersoff potential, which describes the C–C interactions in the calibrated h-FF model, does not implicitly account for changes in coordination number resulting from Cl adsorption. While this description is reasonable for single Cl atom adsorption,^5^ it potentially limits the applicability of the present h-FF model to PCG systems with low Cl content (*X*_Cl_ < 25%), where chlorination induces only small out-of-plane displacements in the graphene lattice, resulting in small variations in the C–C bond angle. For higher *X*_Cl_ levels, larger variations are expected (e.g., DFT calculations predict a reduction in the C–C bond angle from 120° to 115.5° in locally one-sided C_4_Cl configurations^37^).

**Table 2:** C–C and C–Cl Bond Properties Extracted from 1 ns MD Thermalizations of Suspended and Supported PCG Sheets with Low-to-Moderate *X*_Cl_ ^a^

*X*_Cl_ (%)	θ_C–C–C_ (deg)	*d*_C–C_ (Å)	θ_C–C–Cl_ (deg)	*d*_C–Cl_ (Å)	Δ*q* (e)
Suspended PCG sheets					
∼0​^b^	119.18	1.42			
∼3​	119.18	1.42	105.07	2.01	–0.027
∼6​	119.19	1.42	105.39	2.00	–0.026
∼12​	119.12	1.42	106.85	1.96	–0.024
∼25​	119.04	1.42	109.72	1.90	–0.022
PCG sheets supported on Si					
∼1.​5	119.20	1.40	108.02	2.00	–0.027
∼3​	119.20	1.40	107.75	2.00	0.028
∼6​	119.16	1.40	107.18	2.01	–0.028

a The given bond lengths, bond angles, and Δ*q* values are averaged by sampling all the C–C and C–Cl bonds contained in the relaxed PCG systems.

b SLG, opt-Tersoff potential.^24^

The analysis of the *NPT*-relaxed suspended sheets at 300 K (1-ns runs) indicates that the C–C bond length, *d*_C–C_, remains virtually unaffected by chlorination. This finding contrasts with our ground-state MS simulations of PCG sheets, which predict a $d_{C-C}^{0}$ value that increases with increasing *X*_Cl_ (Supplementary Figure S2). Similarly, DFT studies of chlorinated graphene^37^ and fluorographene (i.e., fully fluorinated graphene)^45^ suggest that the relaxed graphene lattice exhibits larger *d*_C–C_ after halogenation. This discrepancy can be attributed to the dynamical relaxation of covalent bonds at finite *T* in our MD simulations, a feature that energy-only first-principles calculations are unable to capture.

While relaxed PCG exhibits sp^2^-type C–C bond angles (θ_C–C–C_) that adhere to the expected 120° (due to minimal buckling), the angles of sp^3^-hybridized C–Cl bonds, θ_C–C–Cl_, display mean values that range from ≈ 105° to ≈ 109° when *X*_Cl_ increases from ∼3% to ∼25%, respectively. (The bond angle is defined as the maximum angle between a bonded C and Cl atom relative to their nearest C atoms.) Bond angles exceeding 100° are also observed in MD simulations of suspended PFG using ReaxFF;^38^ see Supplementary Figure S10c. However, ground-state DFT computations yield θ_C–C–Cl_ = 103° in PCG,^37^ a value ∼4% smaller than the mean θ_C–C–Cl_ calculated from the relaxed sheet with *X*_Cl_ ∼ 6%. This discordance may also stem from thermal effects influencing the relaxation of C–Cl bonds in our MD simulations. Note that θ_C–C–Cl_ is not explicitly constrained by the h-FF model. Supplementary Video provides an atomic-level visualization of the dynamical evolution of Cl atoms around their bonds to C atoms in a relaxed, periodic C_16_Cl cell (300 K).

Although our substrate-supported PCG sheets exhibit statistically larger values of θ_C–C–Cl_ relative to the suspended cases (about 2% increase), we find no significant influence of substrate coupling on the other C–Cl bond properties, including *d*_C–Cl_ and Δ*q*. However, the *d*_C–C_ levels mildly decrease (about 1.4%) with substrate coupling. We attribute this to the restricted out-of-plane displacements hampering microstructure buckling in the supported PCG sheets. Figure 2e displays the relaxed, substrate-supported PCG sheet with *X*_Cl_ ∼ 3%. The corresponding C–Cl bond length and angle distributions are plotted in Figure 2g.

In Table 2, we present the physical and chemical properties of C–C and C–Cl bonds—i.e., bond lengths, bond angles, and Δ*q*—obtained from (suspended and supported) relaxed PCG sheets with various *X*_Cl_ (∼3–25%).

### Phonon Dispersion in PCG Lattices

We evaluate the vibrational modes of various PCG cells by examining the phonon dispersion curves obtained from MD simulations and comparing the resulting phonon spectrum of relaxed PCG with that of SLG. To do so, we run long MD relaxations (10 ns) using SLG and PCG supercells (namely C_16_Cl, C_8_Cl, and C_4_Cl). During the MD runs, the atoms follow the microcanonical *NVE* ensemble while the Langevin thermostat preserves the system’s temperature at the target temperature, *T*. The Langevin thermostat is an effective thermostatting tool to preserve the correct thermal transport properties of crystals when compared with other approaches.^46^ The computational time step (*dt*) is set to 0.2 fs, which ensures a good sampling of all possible vibrational modes of the lattices.^47^

To obtain the phonon dispersion curves from the MD simulations, we utilize the computational technique credited to Kong,^48^ who implemented the calculation of the dynamical matrix in the LAMMPS code (via ‘fix phonon’ command^49^). The dynamical matrix is calculated *on-the-fly* in the frame of perturbation-theory Green’s function in reciprocal space, which requires crystal lattice symmetry. Phonon dispersion is determined from the eigenvalues of the computed dynamical matrices using the PHANA tool^50^ along 3 high symmetry points (Γ, K, M) of the 2D hexagonal honeycomb lattice.^51^

In Figure 3, we plot the phonon dispersion curves of PCG supercells (blue dots) together with the phonon spectrum of SLG (gray lines), corresponding to relaxed sheets at 300 K. The phonon modes of SLG predicted by the opt-Tersoff potential show a decent agreement with those obtained from first-principles calculations. SLG exhibits three acoustic phonon branches, longitudinal acoustic (LA), transverse acoustic (TA), and flexural acoustic (ZA), as well as three optical branches. In suspended SLG, heat transfer is carried mainly by acoustic phonons.^52^ Acoustic phonon diffusion is boosted thanks to a limited phonon–phonon scattering^53^ and decoupled phonon transport of the acoustic modes.^54^ ZA phonons exhibit a quadratic dispersion (around Γ), which differs from the linear dispersion of in-plane TA/LA modes. A symmetry-based selection rule of the graphene lattice results in ZA phonons being only scattered in pairs, restricting phonon scattering processes. As a result, long-lifetime ZA modes are promoted, leading to a high contribution of ZA phonons to the thermal conductivity of SLG.^53,55^

**Figure 3:**
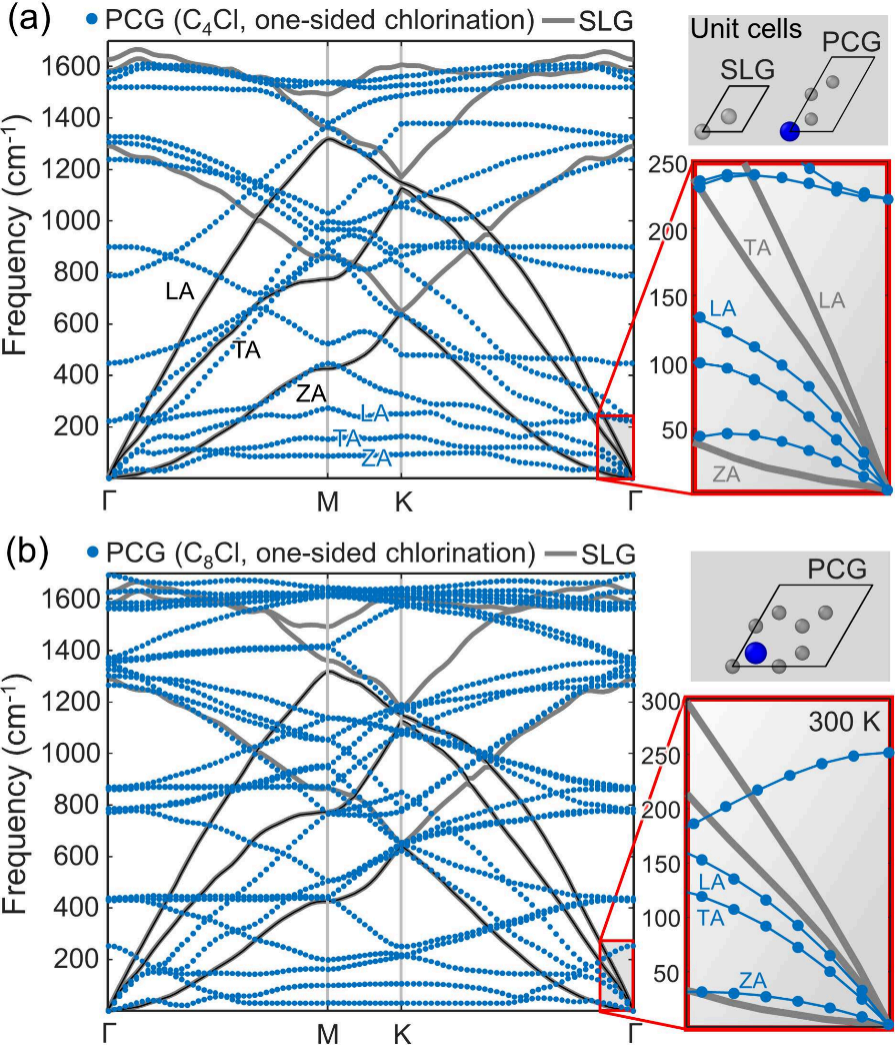
Phonon dispersion in suspended SLG and PCG obtained from MD simulations at *T* = 300 K. The phonon frequencies for (a) C_4_Cl and (b) C_8_Cl supercells (blue points) are drawn together with the spectrum of SLG (gray line). Additional black lines are used to highlight the acoustic modes in SLG (i.e., the ZA, TA, and LA branches). For the analysis, we utilize 12 × 6 C_4_Cl (*X*_Cl_ = 25%), 6 × 6 C_8_Cl (*X*_Cl_ = 12.5%), and 14 × 14 SLG supercells, all containing around 350 atoms. The top insets show the corresponding triclinic unit cells, whereas the bottom insets depict low-frequency, long-wavelength phonon modes near the Γ point, as marked with red squares in the main spectra.

Suppressing the long-wavelength ZA modes, however, results in a marked decrease in thermal conductivity across the SLG lattice. One effective means to induce this effect in graphene is by halogen-atom sp^3^ functionalization. Enhanced scattering processes in the acoustic branches appear to be the underlying mechanism responsible for reducing thermal transport not only in halogenated graphene^55^ but also in 2D carbon allotropes.^56^ Figure 3a shows the phonon dispersion of the C_4_Cl supercell (*X*_Cl_ = 25%), characterized by three acoustic branches and 12 (3*N*_unitcell_ – 3) optical branches. Compared to the highest optical modes at the Γ point in SLG (≈ 1625 cm^–1^, as predicted by the opt-Tersoff potential), the C_4_Cl supercell exhibits a slight downshift (frequency softening) in the highest-frequency modes (≈ 1585 cm^–1^). While the Raman G modes measured by Li et al.^14^ for supported PCG (with increasing *X*_Cl_, up to ∼8%) suggest that G-mode peak frequencies remain unaltered by chlorination; DFT predicts, conversely, significant optical-mode frequency softening in chlorographene^5^ and fluorographene.^39,52,55^ In our PCG cells, optical-mode downshift is only observed for moderate Cl content (C_4_Cl, *X*_Cl_ = 25%), as we find small upshifts in the highest frequency modes (i.e., frequency hardening) of C_8_Cl and C_16_Cl supercells. For example, in the phonon modes of C_8_Cl, the highest-frequency modes upshift to ≈ 1690 cm^–1^ (Figure 3b), whereas similar optical-mode frequencies (≈ 1685 cm^–1^) are obtained in the phonon spectra of C_16_Cl supercells (low Cl content, *X*_Cl_ = 6.25%); see Supplementary Figure S11. In addition, our phonon dispersion results of PCG cells indicate that the slopes of the highest optical modes become markedly flatter (relative to SLG) near the Γ point due to sp^3^ hybridization. In supported SLG, flattening of the highest optical modes has been linked to the suppression of Kohn anomalies due to π-bond hybridization effects.^57^ Accordingly, the vanishing of Kohn anomalies—leading to flat high-frequency optical modes—is also expected in halogenated graphene, as demonstrated in prior DFT calculations of PFG.^39^

Our phonon analysis reveals that the ZA-mode dispersion in PCG remains decoupled from the TA and LA phonons (see the low-frequency modes near Γ in the left insets of Figure 3), which is consistent with the phonon dispersion predicted by DFT for chlorographene^5^ and fluorinated graphene systems.^39,52,55^ The C_4_Cl supercell exhibits a marked frequency softening in the acoustic branches, resulting in frequency levels (at the M and K symmetry points) five to ten times lower than those in SLG. Similar acoustic-mode frequency softening has been reported for C_4_F supercells.^39^ We attribute this acoustic-mode frequency softening to the break of the symmetry selection rule in the graphene lattice due to sp^3^ hybridization, which restricts ZA/LA-TA anharmonicity, thus enhancing phonon scattering. The acoustic frequency levels further soften with low *X*_Cl_, as shown in Figure 3b and Supplementary Figure S11 for C_8_Cl and C_16_Cl supercells, respectively. These outcomes suggest that, in halogenated graphene systems, lower halogen concentrations are associated with a more pronounced softening of acoustic-mode frequencies. Note that all phonon branches obtained from our MD simulations of PCG sheets (C_16_Cl, C_8_Cl, and C_4_Cl supercells) exhibit positive frequencies, further confirming the thermal stability at 300 K of the MD PCG systems.

The phonon frequency modes elucidated by the present h-FF model—tailored for PCG sheets—show a good agreement with those obtained from first-principles calculations and Raman spectroscopy measurements, indicating that our MD scheme can reliably capture the anharmonic lattice dynamics of PCG sheets. Overall, frequency softening in the acoustic modes translates into phonons traveling more slowly, reducing their ability to carry heat across the graphene lattice. These MD outcomes provide a fundamental insight into the effect of chlorination on the thermal conductivity across graphene, which we will discuss in the following section.

### Thermal Conductivity of PCG Sheets

We assess the thermal transport in suspended SLG and PCG sheets with *X*_Cl_ ranging from ∼0.5% to ∼25%. To this end, we perform nonequilibrium molecular dynamics (NEMD) simulations to compute the in-plane thermal conductivity, κ, using the ‘heat-control method’ scheme.^19^ We calculate κ based on Fourier’s heat equation, which correlates the heat-transfer rate flowing throughout a conductive material with the temperature gradient, Δ*T*/Δ*x*, and the cross-sectional area, *A*, through which the heat flows via the following relation:

7 $$\kappa =-J_{x}{\left(\frac{\Delta T}{\Delta x}\right)}^{-1}$$

Here, *J*_*x*_ stands for the (in-plane) heat flux, defined as *J*_*x*_ = Δ*Q*/(2*A* Δ*t*), where Δ*Q*/Δ*t* is the heat current rate. Note that the factor of 2 in the denominator appears because the heat flux is divided into two directions in our NEMD simulations.^58^ Then, the cross-sectional area is *A* = *Ht*, where *t* is the thickness of the sheet. For the SLG sheets, we assume *t* = *t*_G_ = 3.4 Å,^19,20^ while for the PCG sheets, *t* ≈ *t*_G_ + 2*r*_0_ (double-sided chlorination), where *r*_0_ is the C–Cl bond distance imposed by the Morse potential; see Table 1.

The NEMD simulations consist of an initial 50 ps *NVT* run at target *T* that thermalizes the sheets, which are constructed using the *d*_C–C_ value obtained in the relaxed suspended sheets (Table 2). Then, a heat current is induced along the *x* (armchair) direction by subtracting and adding heat to the particles inside two regions (of thickness *w* = 1 nm), which we refer as to the ‘heat sink’ and ‘heat source’, respectively; see the scheme drawn in Figure 4a. The net subtracted/added heat rate is gradually increased from 0 to 1 eV/ps over 500 ps. Then, a constant |Δ*Q*/Δ*t*| of 1 eV/ps is imposed, thereby generating a steady-state temperature gradient in the sheets that runs over 1 ns. The NEMD systems are periodic, allowing for a constant heat flux to flow from the heat source to the heat sink in both senses, as highlighted by the red arrows in Figure 4a. During the heat-transfer (nonequilibrium) stage, the particles follow the microcanonical *NVE* ensemble.

**Figure 4:**
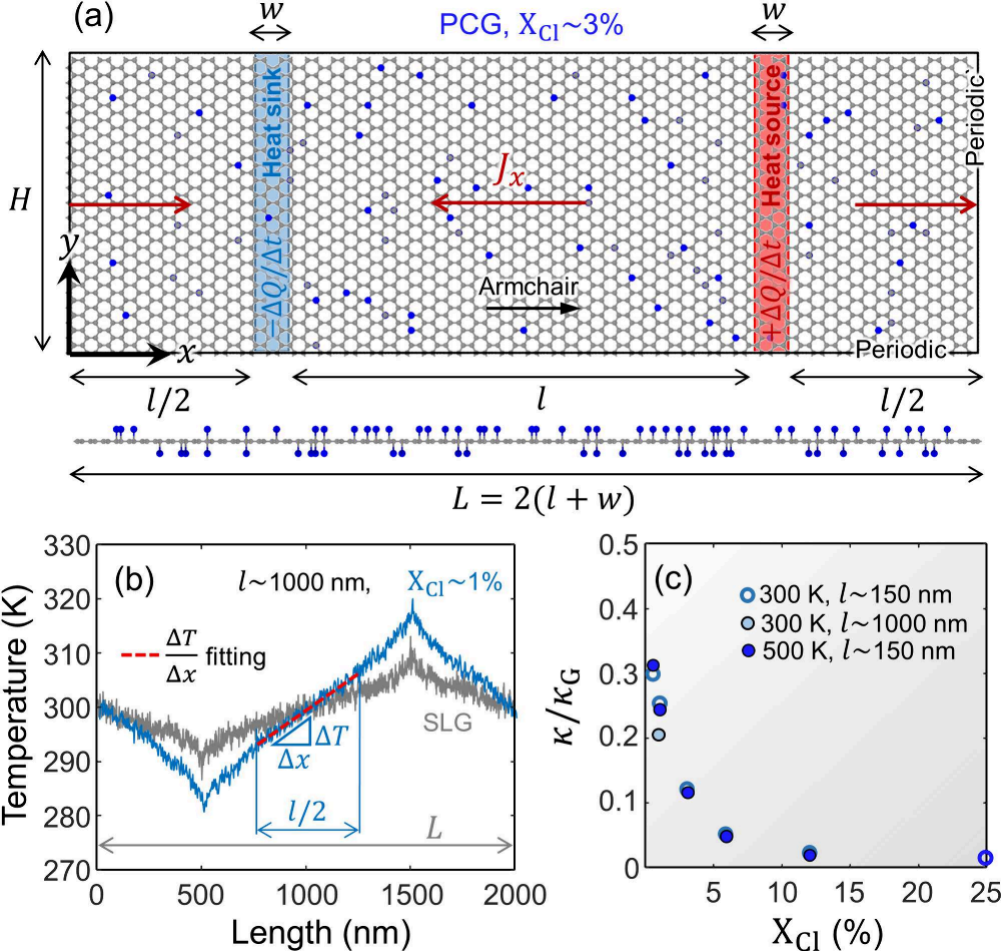
NEMD simulations of SLG and PCG sheets. (a) Schematic representation of a periodic NEMD system containing an as-built PCG sheet (*X*_Cl_ ∼ 3%) of size *L* × *H*. (b) Temperature profile (stationary heat-flux regime) along the *x* axis extracted over 300 ps in SLG (gray line) and PCG with *X*_Cl_ ∼ 1% (blue line) with *L* ≈ 2000 nm. Note that, in this regime, the NEMD simulations maintain constant the imposed heat flux, *J*_*x*_, during which the temperature gradient, Δ*T*/Δ*x*, is measured along a central slot of length *l*/2 (i.e., between the heat source and sink regions), readily allowing the calculation of κ using eq 7. (c) The variation of normalized thermal conductivity, κ/κ_G_, in PCG as a function of *X*_Cl_. κ_G_ stands for the in-plane thermal conductivity of SLG (obtained under the same imposed *T* and *L*).

The MD sheets have a size of *L* × *H* (length, along *x*; height, along *y*; respectively), as depicted in Figure 4a. For the NEMD simulations, we build sheets with *L* ≈ 300 nm and ≈ 2000 nm, and *H* ≈ 10 nm. The number of constituent atoms varies from ∼111.2k–132k atoms in sheets of *L* ≈ 300 nm to ∼750k atoms with *L* ≈ 2000 nm. To obtain the *T* profiles along the sheets, we compute averages of the kinetic energy, *K*, inside consecutive slabs of size 1 nm × *H* from *x* = 0 to *x* = *L*. Then, using the *K* data, the *T*–*x* profile is readily evaluated during the last 300 ps of the NEMD simulation at 10 ps intervals via *T* = 2*K*/(3*k*_B_), where *k*_B_ is Boltzmann’s constant. (Although equilibrium molecular dynamics (EMD) simulations are also commonly used to obtain the value of κ^59^ using the Green–Kubo formalism,^20,60^ recent work by Dong et al.^61^ has shown that the accuracy of both EMD and NEMD methods appears to converge.)

Figure 4b shows the *T* profiles extracted from the NEMD simulation of SLG and PCG (*X*_Cl_ ∼ 1%) with *L* ≈ 2000 nm. Notice in this figure the significant reduction in thermal transport of the graphene lattice when a small amount of Cl atoms (i.e., ∼1% per available C site) bind to SLG to form PCG. We determine Δ*T*/Δ*x* from the linear fitting of the (*x*, *T*) points in a central region of length *l*/2, where *l* is the distance between the heat source and sink regions; see Figure 4a. Finally, the resulting Δ*T*/Δ*x* value is used to compute κ via eq 7.

We compare the κ values of PCG (predicted by the h-FF model) with those of SLG (opt-Tersoff) for various sizes and *T*. Figure 4c shows the normalized thermal conductivity, κ/κ_G_, of PCG sheets with varying *X*_Cl_, where κ_G_ stands for the thermal conductivity of SLG (obtained under similar *T* and *L*). In the PCG sheets, κ/κ_G_ levels abruptly drop with increasing *X*_Cl_. With ultralow Cl content (*X*_Cl_ ∼ 0.5%), κ drops around 3 times relative to κ_G_, following a nonlinear decrease with increasing *X*_Cl_. With moderate Cl content (*X*_Cl_ ∼ 25%), κ further decreases up to around 80 times when compared to SLG. The marked effect of *X*_Cl_ on κ extracted from our simulations align with that reported by Huang et al.,^62^ who conducted NEMD simulations of fluorinated graphene and observed that κ follows a U-shaped trend with increasing F content (*X*_F_) under both random and patterned fluorination, where κ can drop as much as 20 times (relative to SLG) with *X*_F_ ranging between 20% and 70%. Similar κ drops have been predicted for fluorographene (i.e., *X*_F_ = 100%) using first principles.^52,55^

Our NEMD results are in good agreement with previous reports on the length-dependent thermal transport found in graphene sheets, where κ_G_ levels generally rise with sheet length.^63,64^ In our NEMD results, κ_G_ (SLG sheets) increases from ≈ 770 to ≈ 2060 W/(mK) for *l* ∼ 150 and 1000 nm, respectively. A similar trend is observed in the PCG sheets with *X*_Cl_ ∼ 1%, as κ increases from ≈ 193 to ≈ 414 W/(mK) for the same *l* values. With respect to the normalized thermal conductivity, we find that κ/κ_G_ levels mildly decrease when *l* varies from ∼150 nm to ∼1000 nm, as shown in Figure 4c. This suggests that length-dependent thermal transport effects may scale similarly in SLG and PCG sheets, despite the acoustic phonon mean free path being expected to be much smaller in the latter due to the presence of functional sp^3^ groups.

## Conclusions

In this work, we investigate the thermal properties of partially chlorinated graphene (PCG) systems using a hybrid force field (h-FF) framework for classical molecular dynamics (MD) simulations. The present h-FF model combines a Tersoff-type potential for the C–C interactions with pairwise Morse and 12–6 Lennard-Jones potentials for the bonded and nonbonded C–Cl/Cl-Cl interactions, respectively, while incorporating atomic charge equilibration via the QEq method. For the covalent C–Cl bonds, we fit the Morse potential to predict the binding energies and bond lengths reported by density functional theory (DFT) calculations, allowing for an accurate description of key chemical and physical properties of the C–C and C–Cl bonds in chlorinated graphene.

We run MD thermalizations of various PCG systems to evaluate the properties of C–C and C–Cl bonds in relaxed suspended and supported sheets with low-to-moderate Cl content (*X*_Cl_), ranging from ∼1.5% to ∼25%. Also, we compute the phonon dispersion curves of various PCG supercells, comparing the resulting phonon spectra with those of single-layer graphene (SLG). Finally, we assess the effect of chlorination on the thermal properties by performing large-scale nonequilibrium molecular dynamics (NEMD) simulations, which allow us to determine the in-plane thermal conductivity, κ, of suspended SLG and PCG sheets.

The key results of this study are:

1. The h-FF potential tailored for PCG accurately captures fundamental chemical and physical bond properties in suspended and supported sheets, where bond angles and distances follow Gaussian-like distributions and charges of C atoms concentrate around sp^3^-hybridization sites. Our MD outcomes suggest that *X*_Cl_ has a mild effect on C–Cl bond charges and angles. All PCG systems simulated in this study exhibit thermal stability at 300 K.
2. Our phonon analysis indicates that chlorination significantly enhances acoustic-phonon scattering in the graphene lattice, leading to five-to-10-fold downshifts in the acoustic modes of PCG sheets compared to SLG. Additionally, the slopes of the highest optical modes in PCG become noticeably flatter while their frequency levels exhibit only minor shifts.
3. The NEMD results reveal a substantial decrease in normalized thermal conductivity (κ/κ_G_) levels, with a ∼70% reduction with *X*_Cl_ ∼ 1% (relative to SLG). κ/κ_G_ levels further decline to ∼98% with *X*_Cl_ ∼ 25%. This study provides, to the best of our knowledge, the first reported assessment of the thermal conductivity of chlorinated graphene.

Overall, our work demonstrates that the h-FF framework can provide accurate MD predictions of the physical and chemical properties of chlorinated graphene systems, offering critical insights into the influence of chlorination on the thermal transport across the graphene lattice. Nonetheless, further experimental and computational research is necessary to achieve a comprehensive understanding of the thermal and structural properties of chlorinated graphene systems across different chlorination levels and temperatures.

## Supplementary Material







## Notes

S1: Schematic of the MD domains for the thermalizations of systems. S2: Total energy of PCG versus C–C bond distance, obtained from MS simulations. S3: Morse potential fitting to the C–F bond curve predicted by the ReaxFF model. S4: Time evolutions of temperature, box dimensions, and energy during *NPT* thermalizations of SLG and PCG sheets. S5–S7: Binding energy, average charge of Cl atoms, and average charge difference of bonded C–Cl pairs under various Morse and charge-equilibration parameters. S8: Comparative analysis of the computational performance of the h-FF model. S9: Results from MD thermalizations of suspended and supported PCG sheets with *X*_Cl_ ∼ 6%. S10: C–F bond properties in PFG (ReaxFF). S11: Phonon dispersion curves of C_16_Cl supercells (PDF)
(1) LAMMPS input script that runs an *NPT* relaxation of a PCG sheet calling the h-FF potential, (2) LAMMPS read data file of a 7 nm × 7 nm PCG sheet (*X*_Cl_ ∼ 6%), (3) LAMMPS parameter file with the variables for charge equilibration via QEq, (4) the opt-Tersoff potential for graphene,^24^ (5) LAMMPS output file from the MD simulation, and (6) plots of the time evolution of energy, temperature, and box dimensions (ZIP)
Atomic-level visualization of the microstructure in a relaxed, periodic C_16_Cl cell at 300 K (MP4)
