# Supplementary material for: Elucidating the Thermal Properties of Partially Chlorinated Graphene Using Molecular Dynamics Simulations
Source: J Phys Chem C Nanomater Interfaces. 2025 Sep 17;129(39):17767–77. doi: 10.1021/acs.jpcc.5c04046 (PMC12498505; doi:10.1021/acs.jpcc.5c04046)
Supplement: Supplementary file 1 [file jp5c04046_si_001.pdf]

Supporting Information:

"Elucidating the thermal properties of partially chlorinated graphene using molecular dynamics simulations"

Javier Varillas[*,†,‡] and Martin Kalbáč[†]

†*Department of Low-Dimensional Systems. J. Heyrovský Institute of Physical Chemistry, Czech Academy of Sciences, Dolejškova 2155/3, 182 23 Prague 8, Czech Republic.*

‡*Department of Ultrasonic Methods. Institute of Thermomechanics, Czech Academy of Sciences, Dolejskova 1402/5, 182 00 Prague 8, Czech Republic.*

E-mail: javier.varillas@jh-inst.cas.cz

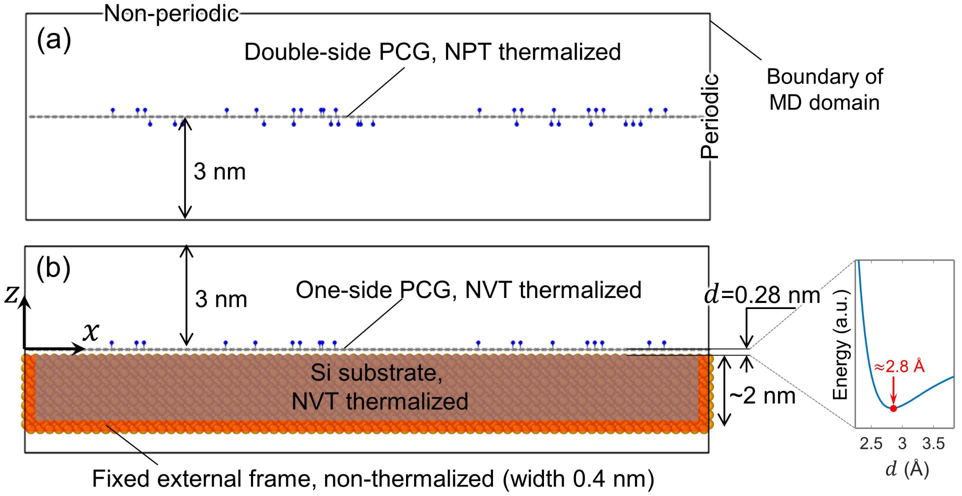

Figure S1: Schematic of the MD domains for the thermalizations of suspended (a) and supported (b) PCG systems. The inset to (b) shows the energy of the supported PCG systems as a function of the separation between the flat PCG sheet and the Si substrate, d, indicating a minimum-energy configuration at $d \approx 2.8$ Å.

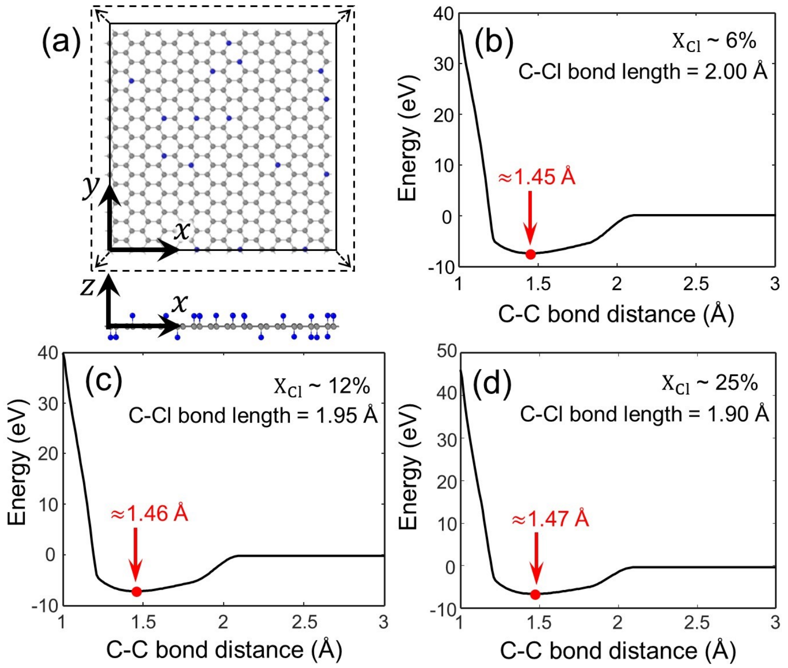

Figure S2: Energy profiles of various PCG sheets with increasing C-C bond distance. The energies were extracted from molecular static (MS) simulations that expanded the simulation domain along the x and y directions, as illustrated in (a). The C-C bond distance was increased from 1 Å to 3 Å, while the C-Cl bond length was maintained fixed at r_0. The (uncharged, flat) PCG sheets contain 336 C atoms, with $X_{Cl} \sim 6\%$ (b), $\sim 12\%$ (c), and $\sim 25\%$ (d). The resulting lowest-energy C-C bond distance is marked with a red arrow in (b-d).

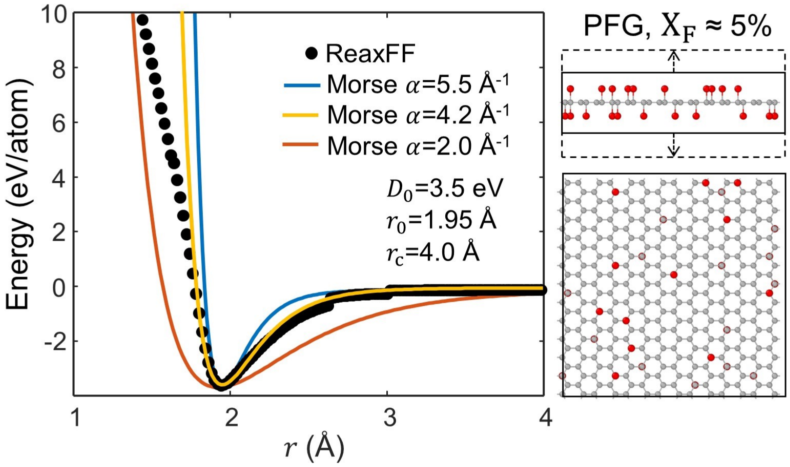

Figure S3: Fitting α of the Morse potential (Equation (5) in the main text) to the C-F bond curve predicted by the ReaxFF model for fluorinated graphene systems.[1] The ReaxFF data points were obtained from an MS simulation that expanded the computational domain along the z direction. The C-F bond distance was increased from 1.5 Å to 4 Å, while the C-C bond length was maintained fixed at 1.452 Å, as predicted by the ReaxFF potential.

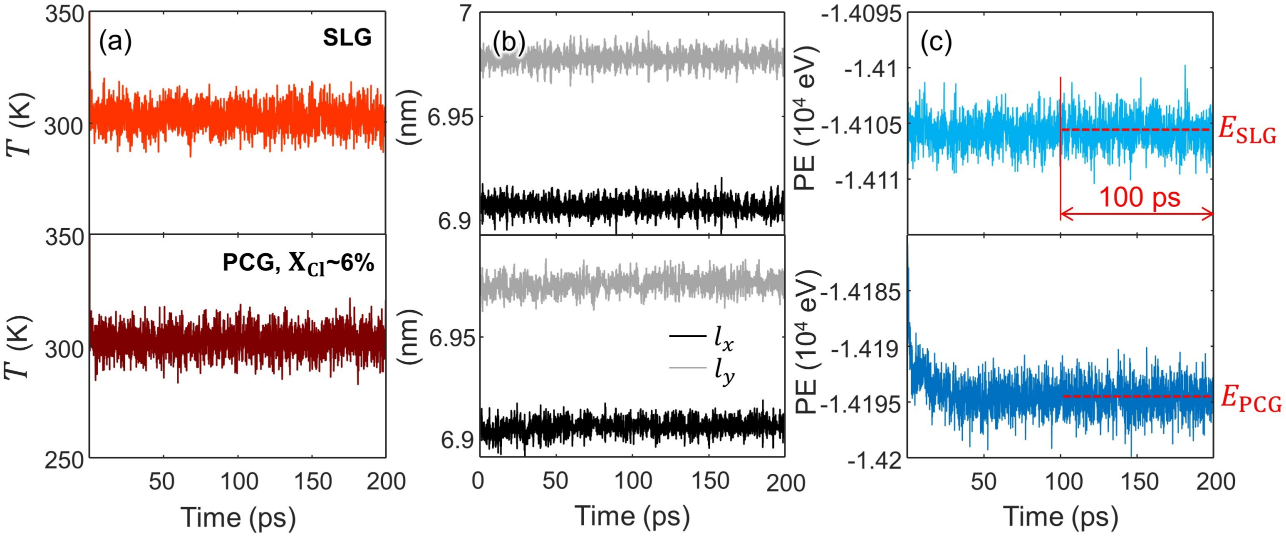

Figure S4: 200-ps NPT thermalizations of SLG (top) and PCG (bottom) sheets. Time evolutions of temperature (T), box dimensions along x and y, and potential energy (PE) are plotted in (a)-(c), respectively.

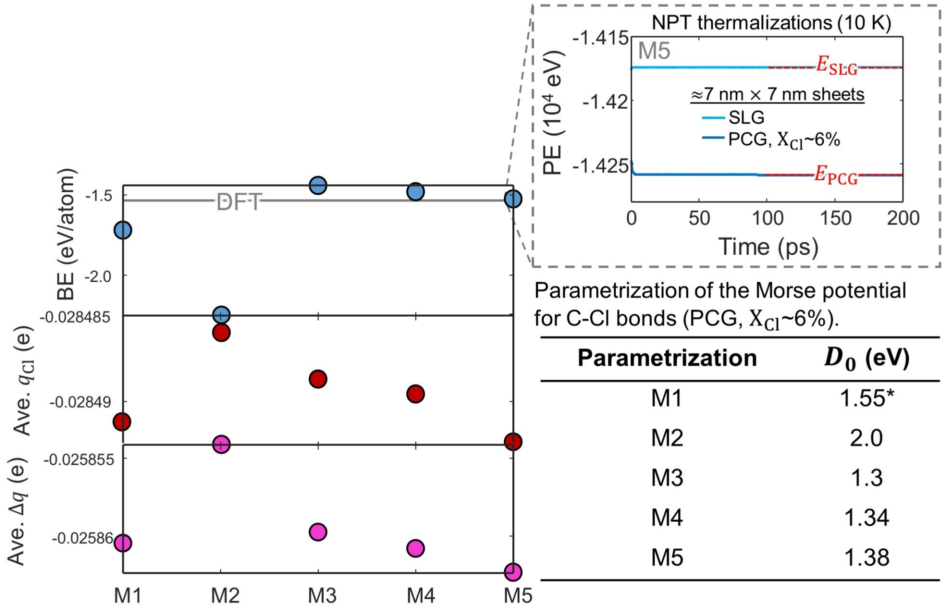

Parametrization of the Morse potential for C-Cl bonds (PCG, X_{Cl}~6%).

Parametrization	D_0 (eV)
M1	1.55*
M2	2.0
M3	1.3
M4	1.34
M5	1.38

Figure S5: Binding energy (BE), average charge of Cl atoms (q_{Cl}), and average charge difference of bonded C-Cl pairs (Δq) obtained in PCG with $X_{Cl} \sim 6\%$ using the Morse parameters M1-M5 (Equation (5) of the main text) given in the right-hand side table. The values for the other Morse parameters (i.e., r_0, r_c, and α) are provided in Table 1 of the main text. The BE, q_{Cl}, and Δq values were extracted from a 200-ns NPT thermalization at 10 K, using periodic sheets of size $\approx$ 7 nm $\times$ 7 nm (Figure S1(a)). *M1 has D_0 value similar to the DFT-predicted BE.[2] For the MD simulations discussed in the main text, we adopted M5. The top inset shows the potential energy (PE) of SLG and PCG sheets (M5 parameters) during thermalization, where PE levels quickly stabilize in both systems. Average PE values E_{SLG} and E_{PCG} (used to determine the BE via Equation (6) of the main text) are calculated during the last 100 ps of the thermalizations, as marked with the dashed red lines.

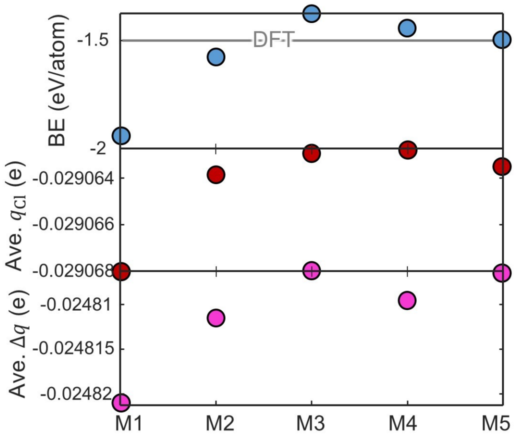

Parametrization of the Morse potential for C-Cl bonds (PCG, X_{Cl}~12 %).

Parametrization	D_0 (eV)
M1	1.5*
M2	1.25
M3	1.1
M4	1.15
M5	1.19

Figure S6: Idem to Figure S2 for $X_{Cl} \sim 12\%$. For the MD simulations discussed in the main text, we adopted M5.

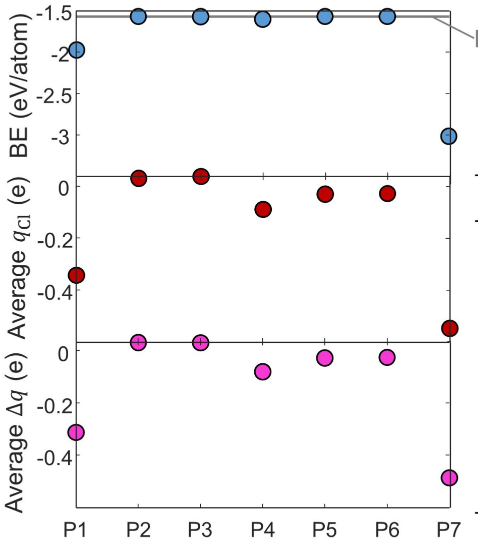

Values of electronegativity (χ) and chemical hardness for C and Cl found in the literature (in eV).

**Parameters**	χ_C	η_C	χ_{Cl}	η_{Cl}
**P1, Ref.[a]**	5.343	10.126	8.564	9.892
**P2, Ref.[b*]**	6.94	12.65	5.99	32.08
**P3, Ref.[b*]**	4.65	11.47	3.45	33.09
**P4, Ref.[b*]**	7.37	10.74	10.19	31.89
**P5, Ref.[c*]**	6.263	10.743	6.602	11.266
**P6, Ref.[c*]**	8.002	10.718	8.426	15.291
**P7, Ref.[d*]**	6.26	4.999	8.29	4.678

Figure S7: Binding energy (BE), average charge of Cl atoms (q_{Cl}), and average charge difference of bonded C-Cl pairs (Δq) obtained in PCG ($X_{Cl} \sim 6\%$, Morse parameters M5; Figure S2) under various charge-equilibration parameters, given in the right-hand side table. Electronegativity, χ, and chemical hardness, η, values for C and Cl isolated atoms are taken from [a]Ref.[3] (P1); [b]Ref.[4] METS (P2), NETS (P3), HETS (P4); [c]Ref.[5] P1 (P5), T1 (P6); and [d]Ref.[6] (P7). The simulations comprised a 200-ns NPT thermalization at 10 K, using sheets of size $\approx$ 7 nm $\times$ 7 nm. For the MD simulations discussed in the main text, we adopted the P5 parameters for charge equilibration (calibration 'T1'; see Table 5 in Ref.[5]). [e]Ref.[2]

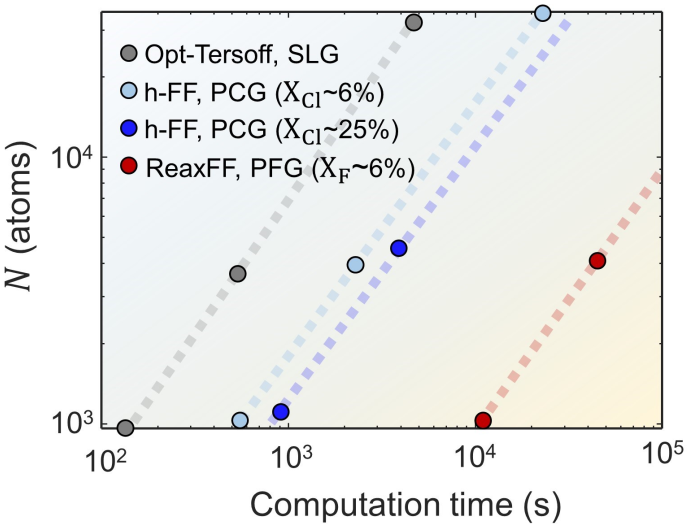

Figure S8: Comparison of computational performance of the h-FF model (for PCG, with timestep $dt = 0.5$ fs) relative to opt-Tersoff (SLG, $dt = 0.5$ fs) and ReaxFF (PFG, $dt = 0.2$ fs). The computation times are obtained from 50-ps NPT thermalization runs at 300 K. We plot the number of atoms (N) vs. computation time (logarithm scale) obtained from 50-ps MD thermalizations of SLG, PCG, and PFC systems (using 1 CPU, Intel Xeon Gold 6226R processor at 2.90 GHz). The dotted lines provide a guide to the eye. We employ MD cells of various sizes containing a number of atoms, N, that ranges from $\sim$ 1k atoms to $\sim$ 35k atoms. Comparative computation times scale equally with the number of CPUs employed using 32 CPUs.

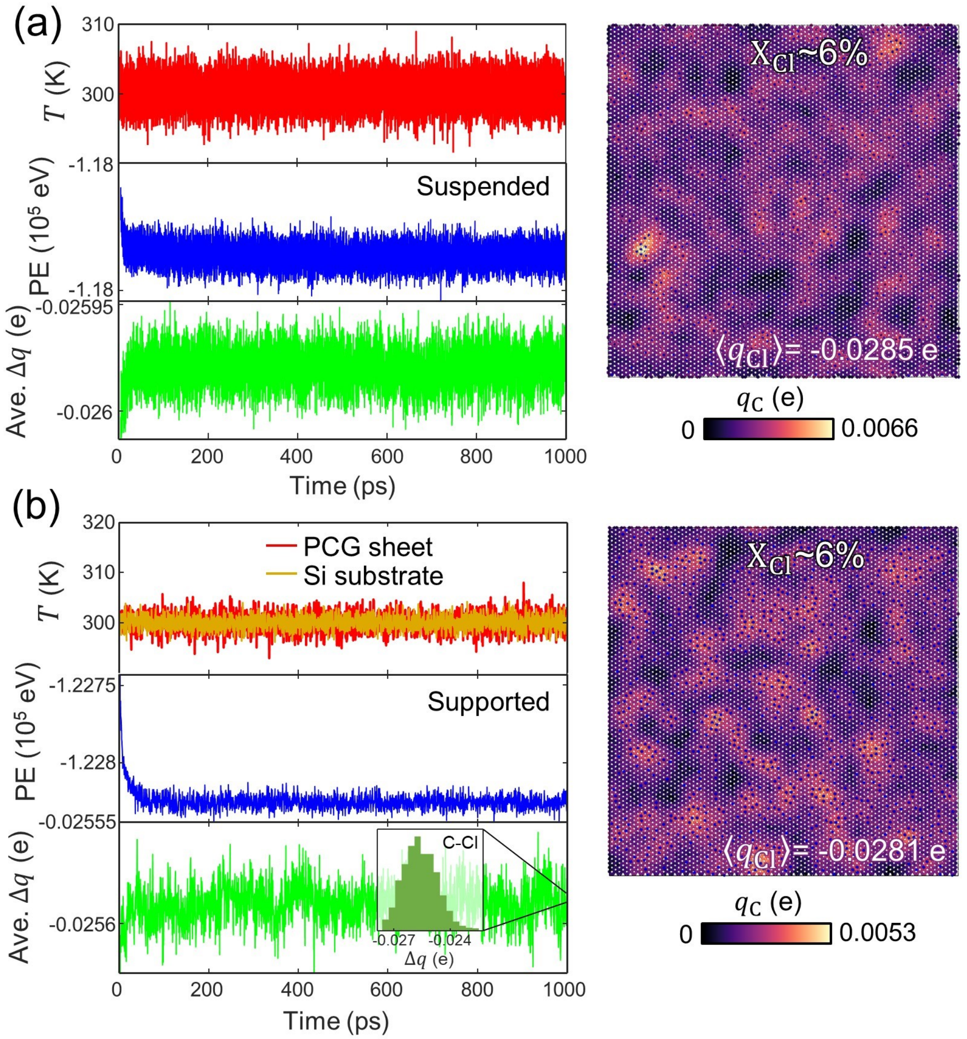

Figure S9: MD thermalizations (over 1 ns) of (a) suspended and (b) supported PCG sheets with $X_{Cl} \sim 6\%$. Left: Temporal evolution of temperature (T), potential energy (PE), and average charge difference in C-Cl bonds (Δq). The inset to (b) shows the distribution of the charge difference values, as evaluated in the 906 C-Cl bonds at 1 ns. Right: QEq-calculated spatial charge distribution in relaxed PCG sheets (i.e., at 1 ns). $\langle q_{Cl} \rangle$ expresses the average value of q_{Cl}.

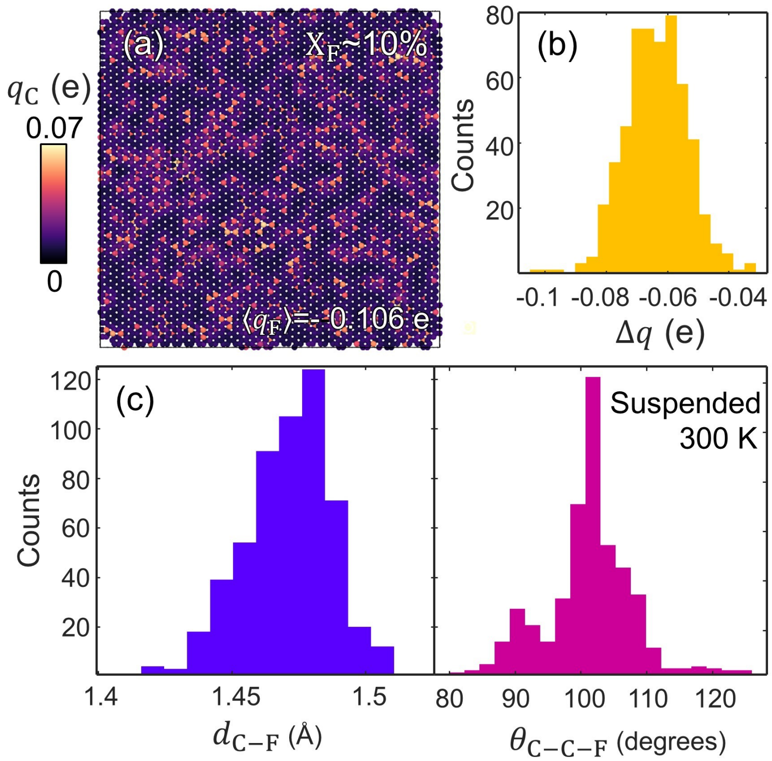

Figure S10: C-F bond properties in PFG with $X_F \sim 10\%$, as predicted by an 0.5-ns NPT thermalization (300 K) using the reactive force field (ReaxFF) model for C-F systems by Sigh et at.[1] The MD cell has a size of ≈ 10 nm $\times$ 10 nm and contains $\sim$ 6k atoms. The timestep is set to 0.2 fs, as the ReaxFF requires a smaller timestep compared to typical force fields.[7] (a) Spatial charge distribution across the C atoms in suspended PFG with $X_F \sim 10\%$ (double-sized fluorination at random C sites). (b) Charge difference distribution between bonded C and F (Δq). The simulation predicts a mean Δq of -0.064 e and a mean q_F of -0.106 e. (c) Distributions of C-F bond distances (d_{C-F}, left) and angles (θ_{C-C-F}, right). Mean d_{C-F} and θ_{C-C-F} values: 1.48 Å and 101.18°, respectively.

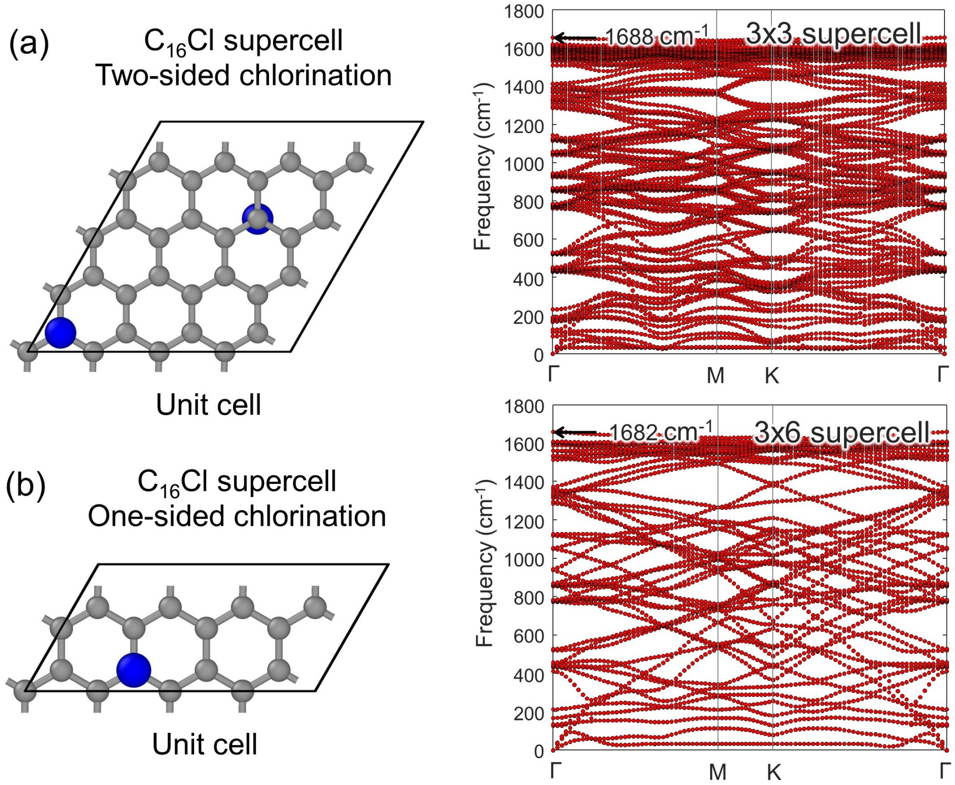

Figure S11: Phonon dispersion curves (right) of $C_{16}Cl$ supercells ($X_{Cl} = 6.25\%$). The phonon spectra for $C_{16}Cl$ supercells with double-sided and single-sided chlorination are given in (a, b), respectively. The left-hand side insets depict the corresponding triclinic unit cells. The supercells are relaxed at $T = 300$ K over 10 ps. Phonon modes are calculated via PHANA.[8] For computational details, see Section 3.2 of the main text.

References

1. Singh, S. K.; Srinivasan, S. G.; Neek-Amal, M.; Costamagna, S.; van Duin, A. C. T.; Peeters, F. M. Thermal properties of fluorinated graphene. *Phys. Rev. B* **2013**, *87*, 104114.

2. Yang, M.; Zhou, L.; Wang, J.; Liu, Z.; Liu, Z. Evolutionary Chlorination of Graphene: From Charge-Transfer Complex to Covalent Bonding and Nonbonding. *J. Phys. Chem. C* **2012**, *116*, 844–850.

3. Rappe, A. K.; Goddard, W. A. I. I. I. Charge equilibration for molecular dynamics simulations. *J. Phys. Chem.* **1991**, *95*, 3358–3363.

4. Verstraelen, T.; Van Speybroeck, V.; Waroquier, M. The electronegativity equalization method and the split charge equilibration applied to organic systems: Parametrization, validation, and comparison. *J. Chem. Phys.* **2009**, *131*, 044127.

5. Verstraelen, T.; Bultinck, P.; Van Speybroeck, V.; Ayers, P. W.; Van Neck, D.; Waroquier, M. The Significance of Parameters in Charge Equilibration Models. *J. Chem. Theory Comput.* **2011**, *7*, 1750–1764.

6. Dong, X.; Oganov, A. R.; Cui, H.; Zhou, X.-F.; Wang, H.-T. Electronegativity and chemical hardness of elements under pressure. *PNAS* **2022**, *119*, e2117416119.

7. van Duin, A. C. T.; Dasgupta, S.; Lorant, F.; Goddard, W. A. ReaxFF: A Reactive Force Field for Hydrocarbons. *J. Phys. Chem. A* **2001**, *105*, 9396–9409.

8. Kong, L. T. Phonon Analyzer (PHANA) algorithm. `https://github.com/lingtikong/phana`, 2021; [Accessed 22-01-2025].
