# Supplementary figures and images for: Elucidating the Thermal Properties of Partially Chlorinated Graphene Using Molecular Dynamics Simulations
Source: J Phys Chem C Nanomater Interfaces. 2025 Sep 17;129(39):17767–77. doi: 10.1021/acs.jpcc.5c04046 (PMC12498505; doi:10.1021/acs.jpcc.5c04046)

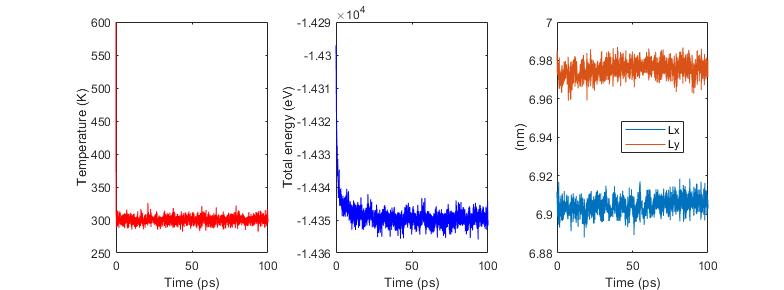

Supplement: Supplementary file 2 [file jp5c04046_si_002.zip › Supplementary_files/outputs.jpg]
